# H is for hypersexual: Sexuality in youths with ADHD

**DOI:** 10.3389/frcha.2022.1048732

**Published:** 2022-11-10

**Authors:** Elijah W. Hale, Molly O. Murphy, Katherine P. Thompson

**Affiliations:** School of Medicine, University of Colorado Anschutz Medical Campus, Aurora, CO, United States

**Keywords:** ADHD, hypersexuality, sexuality disorder, adolescent psychiatry, LGBTQ

## Abstract

**Background:**

Recent research into the association between ADHD and hypersexuality has been extremely skewed toward male patients and contribute to stigma against individuals engaging in same sex sexual behaviors. We sought to expand research on this important relationship and to address these shortcomings.

**Methods:**

Using data from the TriNetX database, we created two patient cohorts of patients 21 years or younger, separated by presence or absence of ADHD diagnosis. We analyzed disorders of sexual behavior, comorbid psychiatric illness, and medication type. Those with ADHD were subdivided into same-sex high risk sexual behavior (HRSB) and opposite-sex HRSB. The ADHD group was analyzed based on the presence or absence of any disorder of sexuality. The outcomes measured were disorders of sexual behavior, comorbid psychiatric illness, and medication type.

**Results:**

In a study of 1,355,184 analyzed patient records, patients ADHD were more likely to have all measured outcomes. Orientation of HRSB did not impact for disorders of sexual behavior, but individuals with same-sex HRSB were at higher risk for comorbid psychiatric illness. Those with hypersexuality and ADHD were a higher risk for psychiatric illnesses.

**Conclusion:**

Our study is the largest to date examining associations between hypersexual behaviors and ADHD. Increased awareness of this association may aid in addressing some problematic behaviors before they become detrimental to the individuals with ADHD or others within their lives.

## Introduction

Attention-Deficit/Hyperactivity Disorder (ADHD) is a neurodevelopmental disorder affecting over 5% of youth in the United States [1]. Though diagnosis focuses on an extended period of disruptive hyperactive-impulsive and/or inattentive symptoms attention, ADHD has also been associated with an increased risk for obesity, sleep problems, sexually transmitted infections, immune disorders, metabolic disorders, and other health risks [2]. While many of these associations are the focus of extensive research, hypersexuality, and other disorders of sexuality among individuals with ADHD remain a poorly understood and potentially significant detriment to patients' quality of life.

Hypersexuality describes an inability to regulate one's sexual behavior with significant personal distress [3]. Hypersexuality and other disorders of sexuality involve a state of arousal that temporarily and adversely impact cognitive processing, leading to patterns of behavior that may be incongruent with normophilic behaviors [4].

Importantly, hypersexuality and other compulsive sexual disorders are resistant to treatment, and present with patterns seen in addictive disorders, leading to much debate around how to classify these differences in sexual behavior [5]. As ADHD is known to affect behavior and impulsivity, the association between hypersexuality and ADHD is an area of frequent discussion.

Previous studies on hypersexuality among ADHD populations have been both limited in scope and potentially stigmatizing. Study populations should be diverse and representative, not unduly focused on sex offenders, LGBTQ+ individuals, or males, in order to avoid perpetuating harmful stereotypes and excluding potentially impacted groups. Additionally, disorders of sexuality associated with ADHD should be intentionally studied among adolescents, as this developmental period is notable for heightened sexual discovery and is therefore at elevated risk of experiencing symptoms of inappropriate hypersexuality [6]. We had two novel hypotheses: we would find no difference based on sexual orientation, and we would find associations between hypersexuality and psychiatric comorbities and stimulant medications. In addition to these hypotheses, our study aims to rectify some oversights in previous analyses and provide both the largest single study to date of this patient population, particularly limited to the period of adolescence.

## Methods

This study utilized deidentified, aggregate data obtained from the TriNetX database, which contains electronic medical records from large healthcare organizations and has shown utility for a variety of medical fields including cancer and infectious disease [7, 8]. TriNetX, LLC is compliant with the Health Insurance Portability and Accountability Act (HIPAA), the US federal law which protects the privacy and security of healthcare data, and any additional data privacy regulations applicable to the contributing HCO [9]. TriNetX is certified to the ISO 27001:2013 standard and maintains an Information Security Management System (ISMS) to ensure the protection of the healthcare data it has access to and to meet the requirements of the HIPAA Security Rule. Any data displayed on the TriNetX Platform in aggregate form, or any patient level data provided in a data set generated by the TriNetX Platform only contains de-identified data as per the de-identification standard defined in Section §164.514(a) of the HIPAA Privacy Rule [9].

Using International Classification of Diseases (ICD) codes, we created two patient cohorts. All patients were at most 21 years old, with one group having a diagnosis of ADHD (F90) and the other not. We also separated the ADHD cohort for two separate sub-analyses. One sub-analysis separated patients with ADHD based on presence of same-sex high risk sexual behavior (HRSB, ICD: Z72.52-3) or presence of opposite-sex HRSB (Z72.1). The other sub-analysis separated patients with ADHD based on the presence or absence of any disorder of sexuality, further defined below.

The studied outcomes fell into three categories: disorders of sexual behavior, comorbid psychiatric illness, and medication type. Disorders of sexual behavior included: high risk sexual behavior (HRSB), same sex HRSB, opposite sex HRSB, paraphilia disorders, “other sexual disorder”- often increased libido, compulsive sexual disorder, or “hypersexual disorders” which included all of the previous diagnoses. Comorbid psychiatric illnesses included: depressive episode or major depressive disorder (MDD); anxiety, stress-related, or other non-psychotic disorders; and substance use disorder. Finally, medications were separated by amphetamine-class medications and phenidate-class medications.

Using the TriNetX software, we performed 3 sets of statistical analysis. One analysis was between the cohort with ADHD and the cohort without, and the other two analyses separated the ADHD cohort by the characteristics described above, opposite vs. same sex behavior and presence vs. absence of any hypersexuality disorders. Prior to comparison, the cohorts were balanced based on age, sex, ethnicity, and race using nearest-neighbor matching to a difference in propensity scores <0.1 [7]. After matching, cohorts had no significant differences in sex, age, ethnicity, or race. Table 1 contains cohort demographic information for the non-ADHD and overall ADHD cohorts, both before and after balancing on these characteristics. Balancing was performed before each of the three analyses. A *t*-test was used to compare event rates between cohorts. Relative risk and odds ratios with a 95% confidence interval were also calculated from event rates. The results are compiled in Table 2. Significance for this study was set at *p* < 0.05. As this study contained only deidentified aggregate data, the Colorado Multiple Institutional Review Board (COMIRB) designated it as non-human research not in need of approval.

**Table 1:** Demographics of overall ADHD cohort and not ADHD reference cohort, before and after propensity score matching on ethnicity, sex, race, and age.

**Demographic**	***N***	**% of cohort**	***P*-value**	**Demographic**	***N***	**% of cohort**	***P*-value**
**(Pre-matching)**				**(Post-matching)**			
**Current age**	**(Mean** **+/– SD)**			**Current Age**	**(Mean** **+/– SD)**		
Not ADHD	15,216,980	11.7 ± 5.94	<0.001	Not ADHD	677,592	14.6 ± 4.19	1
ADHD—overall	683,274	14.6 ± 4.19		ADHD—overall	677,592	14.6 ± 4.19	
**Male**				**Male**
Not ADHD	7,739,892	51%	<0.001	Not ADHD	460,351	68%	1
ADHD—Overall	460,351	68%		ADHD—Overall	460,351	68%	
**Female**				**Female**
Not ADHD	7,450,370	49%	<0.001	Not ADHD	217,047	32%	1
ADHD—overall	217,047	32%		ADHD—overall	217,047	32%	
**White**				**White**
Not ADHD	7,646,730	50%	<0.001	Not ADHD	417,158	62%	1
ADHD—overall	417,158	62%		ADHD—overall	417,158	62%	
**Black**				**Black**
Not ADHD	2,443,597	18%	<0.001	Not ADHD	125,094	18%	1
ADHD—overall	125,094	16%		ADHD—overall	125,094	18%	
**Hispanic/Latino**				**Hispanic/Latino**
Not ADHD	2,425,180	16%	<0.001	Not ADHD	72,187	11%	1
ADHD—overall	72,187	11%		ADHD—overall	72,187	11%	
**Not Hispanic/Latino**				**Not Hispanic/Latino**
Not ADHD	7,473,115	49%	<0.001	Not ADHD	453,704	67%	1
ADHD—overall	453,704	67%		ADHD—overall	453,704	67%	
**Unknown race**				**Unknown race**
Not ADHD	4,564,902	30%	<0.001	Not ADHD	123,940	18%	0.05
ADHD—overall	123,940	18%		ADHD—overall	120,540	17%	
**Unknown ethnicity**				**Unknown ethnicity**
Not ADHD	5,318,685	35%	<0.001	Not ADHD	151,708	22%	0.05
ADHD—overall	151,708	22%		ADHD—overall	153,395	23%	

Bolded values indicate P < 0.05.

**Table 2:** Event statistics by cohort including cohort *N*, outcome *N*, absolute risk, relative risk, odds ratio with 95% CI, and *t*-test *p* value.

**Event (ICD 10)**	**Cohort *N***	**Event *N***	**Risk**	**Rel. risk**	**Odds ratio (95% CI)**	***P* value**	**Event, (ICD 10)**	**Cohort *N***	**Event *N***	**Risk**	**Rel. risk**	**Odds ratio (95% CI)**	***P* value**
*Any hypersexual disorder (F65, Z72.5, F66, F52.8)*							*Paraphilia disorders (F65)*						
ADHD	677,592	7,806	1.15%	**469%**	**4.734**	**<0.001**	Same sex HRSB*	635	10	1.57%	100%	1	1
No ADHD	677,592	1,664	0.25%		(4.49, 4.992)		Opposite sex HRSB*	635	10	1.57%		(0.413, 2.419)	
*High risk sexual behavior (Z72.5)*							“*Other sexual disorder,” Often increased libido (F66)*						
ADHD	677,592	6,366	0.94%	**415%**	**4.183**	**<0.001**	Same sex HRSB*	635	11	1.73%	92%	0.915	0.83
No ADHD	677,592	1,533	0.23%		(3.955, 4.423)		Opposite sex HRSB*	635	12	1.89%		(0.401, 2.09)	
*High risk opposite sex behavior (Z72.51)*							*Hypersexuality/compulsive sexual disorder (F52.8)*						
ADHD	677,592	5,118	0.76%	**377%**	**3.795**	**<0.001**	Same sex HRSB*	635	10	1.57%	100%	1	1
No ADHD	677,592	1,356	0.20%		(3.575, 4.03)		Opposite sex HRSB*	635	10	1.57%		(0.413, 2.419)	
*High risk same sex behavior (Z72.52-53)*							*Depressive episode or MDD (F32, F33)*						
ADHD	677,592	1,332	0.20%	**663%**	**6.638**	**<0.001**	Same sex HRSB*	635	429	67.6%	**131%**	**1.949**	**<0.001**
No ADHD	677,592	201	0.03%		(5.723, 7.699)		Opposite sex HRSB*	635	328	51.7%		(1.552, 2.448)	
*Paraphilia disorders (F65)*							ADHD + Hypersexuality	7,806	4,277	54.8%	**201%**	**3.236**	**<0.001**
ADHD	677,592	798	0.12%	**1353%**	**13.540**	**<0.001**	ADHD - Hypersexuality	7,806	2,127	27.2%		(3.027, 3.46)	
No ADHD	677,592	59	0.01%		(10.394, 17.639)		*Anxiety, dissociative, stress-related, and other nonpsychotic mental disorders (F40-48)*						
“*Other sexual disorder,” often increased libido (F66)*							Same sex HRSB*	635	529	83.3%	**131%**	**2.834**	**<0.001**
ADHD	677,592	628	0.09%	**872%**	**8.729**	**<0.001**	Opposite sex HRSB*	635	405	63.8%		(2.177, 3.69)	
No ADHD	677,592	72	0.01%		(6.84, 11.141)		ADHD + Hypersexuality	7,806	5,273	67.6%	**154%**	**2.678**	**<0.001**
*Hypersexuality/compulsive sexual disorder (F52.8)*							ADHD – Hypersexuality	7,806	3,414	43.7%		(2.509, 2.858)	
ADHD	677,592	337	0.05%	**1,465%**	**14.659**	**<0.001**	*Substance use (alcohol, opiate, cannabis, sedative, cocaine, stimulants, hallucinogens…)*						
No ADHD	677,592	23	0.003%		(9.608, 22.364)		Same sex HRSB*	635	321	50.6%	**168%**	**2.376**	**<0.001**
*Depressive episode or MDD (F32, F33)*							Opposite sex HRSB*	635	191	30.1%		(1.888, 2.991)	
ADHD	677,592	126,520	18.7%	**573%**	**6.814**	**<0.001**	ADHD + Hypersexuality	7,806	2,717	34.8%	**375%**	**5.215**	**<0.001**
No ADHD	677,592	22,087	3.3%		(6.714, 6.915)		ADHD – hypersexuality	7,806	725	9.3%		(4.768, 5.703)	
*Anxiety, dissociative, stress-related, and other nonpsychotic mental disorders (F40-48)*							*Amphetamine-type Stimulant Medications*						
ADHD	677,592	261,321	38.6%	**572%**	**8.682**	**<0.001**	ADHD + Hypersexuality	7,806	2,993	38.3%	**135%**	**1.561**	**<0.001**
No ADHD	677,592	45,692	6.7%		(8.589, 8.775)		ADHD – Hypersexuality	7,806	2,224	28.5%		(1.46, 1.669)	
*Substance use (alcohol, opiates, sedative, cocaine, hallucinogens…)*							*Methylphenidate-type Stimulant Medications*						
ADHD	677,592	43,897	6.5%	**437%**	**4.605**	**<0.001**	ADHD + Hypersexuality	7,806	3,092	39.6%	**124%**	**1.390**	**<0.001**
No ADHD	677,592	10,041	1.5%		(4.505, 4.708)		ADHD – Hypersexuality	7,806	2,503	32.1%		(1.301, 1.484)	

Bolded values indicate statistical significance for intergroup comparison. ^*^HRSB, High Risk Sexual Behavior (Z72.5).

## Results

**Figure 1:**
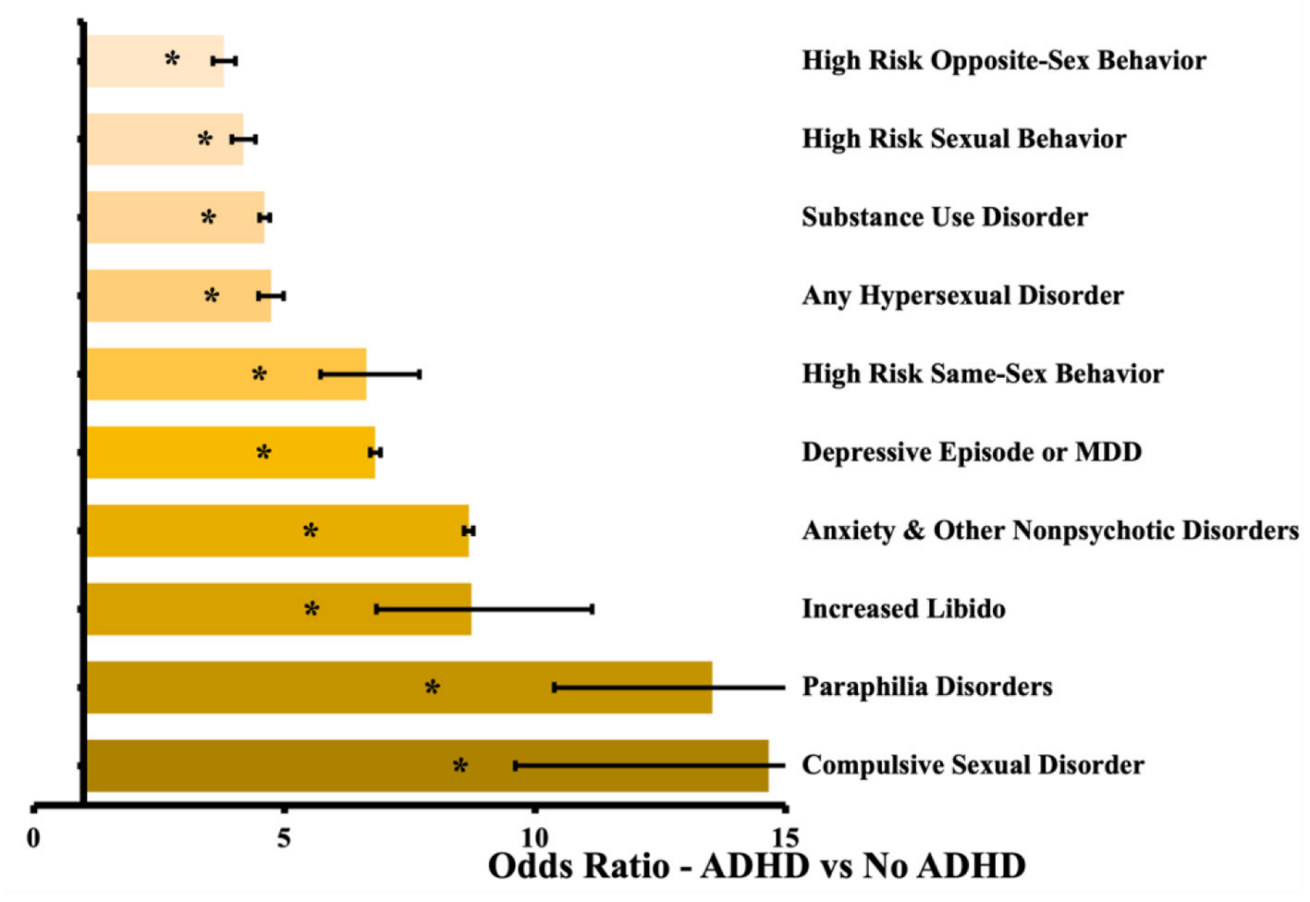
Odds ratio by outcome between patients without ADHD and with ADHD. Confidence bars represent 95% interval. Asterisk (^*^) data labels indicate significant difference (*P* < 0.05).

**Figure 2:**
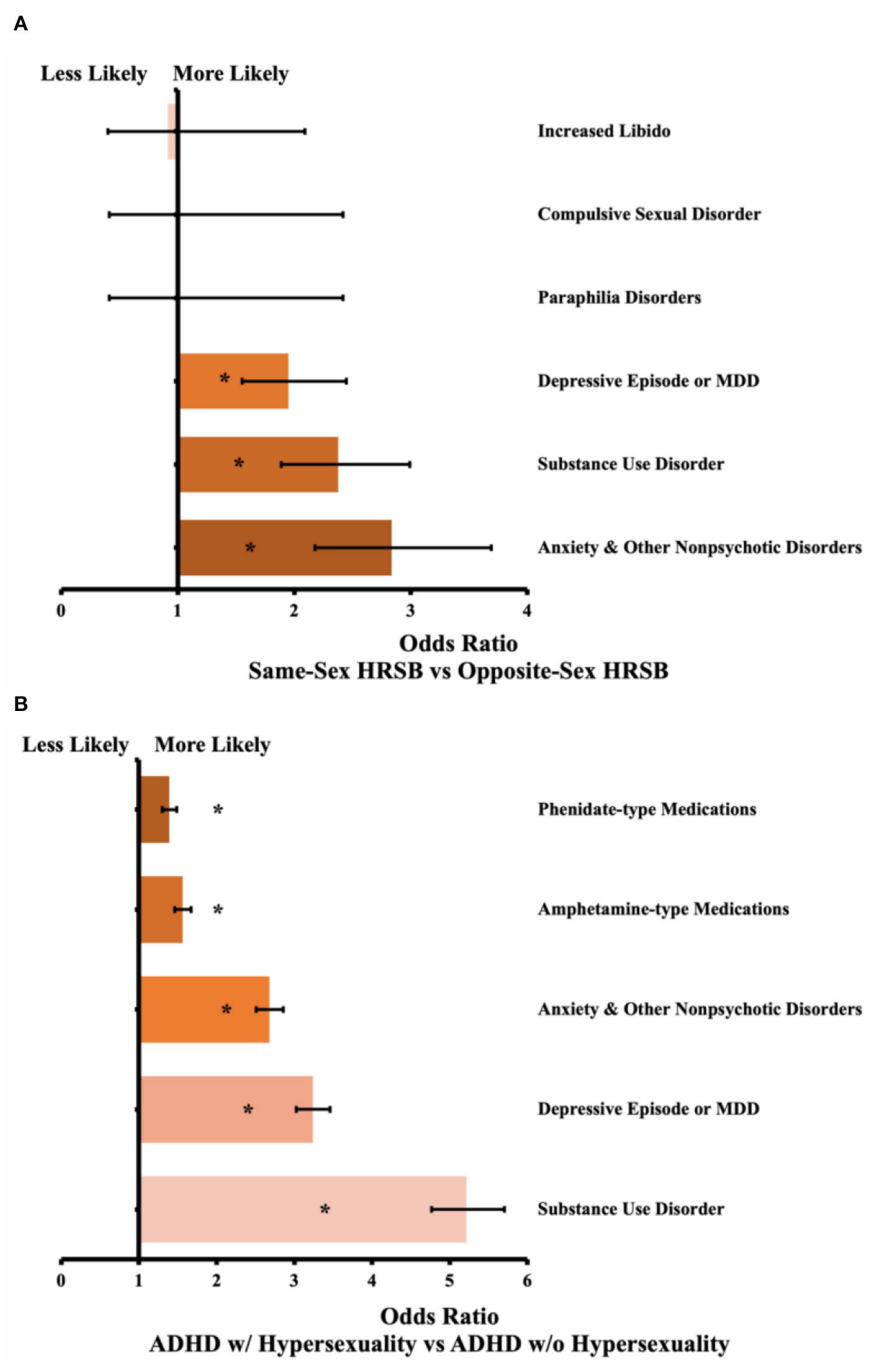
**(A)** Odds ratio by outcome between patients with same-sex vs. opposite-sex HRSB. Confidence bars represent 95% interval. *N* = 635 pairs, asterisk (*) data labels indicate significant difference (*P* < 0.05). **(B)** Odds ratio between patients with ADHD and hypersexuality vs. ADHD without hypersexuality. Confidence bars represent 95% interval. *N* = 7,806 pairs, asterisk (*) data labels indicate significant difference (*P* < 0.05).

We identified 683,274 patients under 21 with an ADHD diagnosis, and we matched these patients to another 677,592 patients without ADHD as described above, for a total of 1,355,184 analyzed patient records. Of the patients with ADHD, we analyzed 635 pairs of patients with HRSB, and 7,806 pairs were included in the presence vs. absence of hypersexuality sub-analysis. Between the ADHD and non-ADHD groups, every measured outcome was more likely to occur in patients with ADHD. The differences ranged from 3.8 times more likely for HRSB with the opposite sex, to 14.7 times more likely for compulsive sexual disorder. The sub-analysis based on HRSB did not show significant differences for disorders of sexual behavior, but did show an increased likelihood for the identified comorbid psychiatric illnesses. The sub-analysis based on hypersexuality within ADHD showed significant increased likelihood for all psychiatric illnesses and both medication types. Further results are in Table 2, along with odds ratios and confidence intervals. Results of the main ADHD analysis is presented graphically in Figure 1. The sub-analysis based on HRSB is presented in Figure 2A, and the sub-analysis based on hypersexuality is presented in Figure 2B.

## Discussion

Our study reveals a large difference in risks for all outcomes between patients with and without ADHD. From an overarching view, patients with ADHD had a nearly 5-times higher rate of any hypersexual disorder, which is in-line with some prior research [10]. However, our associations are present after matching on age, sex, race, and ethnicity, and provide a more representative view of the overall ADHD community; for example, our study included 68% male patients, in comparison to prior studies consisting of more than 90% male patients [11]. We also included far more patients than any previous research, including more than 675,000 matched pairs of patients. Therefore, it is likely that our findings of increased likelihood for all events is a true association and should be addressed.

Our sub-analyses provide novel findings on the comorbidity of hypersexual disorders with other psychiatric illness, as well as the impact of same-sex vs. opposite-sex sexual behavior. Unfortunately, prior research into hypersexuality within ADHD has frequently reinforced stigma around men having sex with men (MSM), both through implying causation between their MSM status and their sexual disorder [12], and through confounding bias in participant selection, such as selecting only MSM sex offenders [13]. The resulting impact is a pseudoscientific appearance that MSM with ADHD have an increased rate of sexual disorders. By analyzing two ADHD sub-groups with either same-sex or opposite-sex behavior, we can challenge the stigmatizing results of some previous studies. In our findings, the orientation of HRSB was not associated with an increased risk of any studied disorders of sexual behavior. It was associated with an increased risk of depression, anxiety or stress-related disorders, and substance use disorder. Those associations are in line with prior research on MSM individuals [14], which further supports the validity of our results and may point toward potential interventions within this group.

Beyond the utility in reducing stigma and providing methodologically rigorous findings, our data holds important significance for any medical professional interacting with ADHD youth. Many of the sexual disorders studied represent significant harm for both the individual with ADHD and their partners in intimate relationships [15]. Certain disorders, such as paraphilia disorders, commonly progress into illegal behaviors, which can cause substantial disruption of one's life, and are often resistant to treatment and therefore should be prevented whenever possible [16]. Our study focuses on individuals 21 years of age or younger, which represents a common period of sexual exploration and development in the United States [17]. Perhaps the simplest intervention supported by our data is an increased awareness and further discussion as individuals with ADHD progress through their sexual development. While it may be unfeasible that primary care providers take a substantial portion of their visits to delve into specifics of sexual behavior, an increased awareness around these issues could allow providers to make appropriate referrals to qualified therapists. Psychotherapy has proven useful in preventing development and progression of many of the studied outcomes, such as depression, anxiety, as well as compulsive sexual behaviors and paraphilia disorders [18].

The positive impact of initiating conversations around sexual behavior and sexual development cannot be understated [19]. ADHD is wellknown to interfere with social development in a variety of domains, which can lead to individuals with ADHD engaging in risky behavior without the social capacity to explore their thoughts and feelings around those behaviors [20]. This presents a self-feeding cycle that cannot be broken by the individual with ADHD alone. Primary care providers, psychiatrists, and psychologists should be frequently asking about and offering resources regarding sexuality, sexual behaviors, and sexual development, particularly in relation to medications. Within our study, both classes of stimulants were associated with hypersexuality; unfortunately the causal link is impossible to establish, as it is conceivable that the increased mental arousal from stimulants could lead to more sexual behaviors [21], or that individuals requiring stimulants are simply more symptomatic, which is supported by prior research on ADHD overall [22]. Physicians managing ADHD with medication should check in with their patients regarding changes in sexual behaviors following medication adjustment, and should include these considerations in the selection of medication regimen.

Ultimately, our study provides conclusive evidence there is a strong association between ADHD and a variety of disorders of sexual behavior, and that the association seems to be present at similar rates for individuals engaging in same-sex behavior compared to those engaging in opposite-sex behavior. It also confirms that many disorders of sexual behavior are present in disproportionate amounts within ADHD prior to age 22, which indicates a strong window of opportunity for intervention. Each year, more individuals are first diagnosed with ADHD in college or university [23], which also corresponds to a time of sexual exploration for many [24]. Physicians involved in ADHD care in university health clinics should use our data to re-evaluate their patient base and consider how often sexual behavior or development is being discussed during ADHD visits, particularly during the critical age range present on college campuses. Similarly, family medicine and child psychiatrists may find utility in our data as a reminder to forewarn and pre-emptively discuss these issues with younger patients, and ensure they have sufficient support, whether in the form of discussions with that physician or use of a therapist. Disorders of sexual behavior can be extremely difficult to treat once they have taken hold in an individual's psyche, and thus any opportunity to prevent development in the first place should be taken [25].

By performing matched pair balancing based on age, sex, ethnicity, and race using nearest-neighbor matching to a difference in propensity scores <0.1, we were able to avoid many confounding variables that hampered prior research into this topic. Similarly, our study addresses large gaps in the research by not limiting our study to individuals registered as sex offenders, or only gay men, which unfortunately represent a disproportionate number of prior studies into ADHD and sexual behavior [11, 26]. However, our study does have limitations based on the data used. As described above, we used HRSB as a partial proxy for sexual orientation; while it is conceivable that some individuals who discuss their same-sex sexual experience with medical professionals would consider themselves to be straight, the analysis performed provides utility in reducing stigma for this research topic. Similarly, our analyses are likely underestimates, as we limited our measurements to ICD codes. As providers are unlikely to enter an ICD code for a non-existing diagnosis but may neglect to enter an ICD code for a diagnosis that does exist, our outcomes are likely a lower limit on the true prevalence. Finally, the age of our patients is both a strength and a potential limitation; sexual behaviors may not present fully prior to age 21, but prior research has indicated problematic behaviors often take root in adolescence [27]. Therefore, by restricting our age limit to 21, we are focusing on youths with ADHD that may benefit the most from specific interventions around sexual behaviors.

Our study is the largest to date examining associations between hypersexual behaviors and ADHD. It is also unique in the age range studied and the sub-analyses performed by both orientation of sexual behavior and presence vs. absence of hypersexual disorders. Overall, there is a clear and large difference in prevalence of disorders of sexual behavior in youths with ADHD, which is important for psychiatrists, primary care physicians, and psychologists. Increased awareness of this association may aid in addressing some problematic behaviors before they become detrimental to the individuals with ADHD or others within their lives. Finally, our study provides useful data that is far more generalizable than prior studies, due to differences in subject selection as described above. It is our hope that our study will call further attention to this important issue within ADHD care, and potentially increase the support available for youths experiencing differences in sexual behavior.

## Data availability statement

The original contributions presented in the study are included in the article/supplementary material, further inquiries can be directed to the corresponding author.

## Ethics statement

As this study contained only deidentified aggregate data, the Colorado Multiple Institutional Review Board (COMIRB) designated it as non-human research not in need of approval.

## Author contributions

EH served as first author with primary contributions to study design and manuscript creation, and had full access to the data used in the study. MM and KT served as co-authors with equal contribution to manuscript revision, statistical support, and other editorial contributions. All authors contributed to the article and approved the submitted version.

## Conflict of interest

The authors declare that the research was conducted in the absence of any commercial or financial relationships that could be construed as a potential conflict of interest.

## Publisher's note

All claims expressed in this article are solely those of the authors and do not necessarily represent those of their affiliated organizations, or those of the publisher, the editors and the reviewers. Any product that may be evaluated in this article, or claim that may be made by its manufacturer, is not guaranteed or endorsed by the publisher.
